# Structural insights into DNA recognition by the BEN domain of the transcription factor BANP

**DOI:** 10.1016/j.jbc.2023.104734

**Published:** 2023-04-20

**Authors:** Ke Liu, Jin Zhang, Yuqing Xiao, Ally Yang, Xiaosheng Song, Yanjun Li, Yunxia Chen, Timothy R. Hughes, Jinrong Min

**Affiliations:** 1 Hubei Key Laboratory of Genetic Regulation and Integrative Biology, School of Life Sciences, Central China Normal University, Wuhan, PR China; 2 Donnelly Centre for Cellular and Biomolecular Research, University of Toronto, Toronto, Ontario, Canada; 3 Structural Genomics Consortium and Department of Physiology, University of Toronto, Toronto, Ontario, Canada

**Keywords:** BEN domain, BANP, BEND6, DNA methylation, X-ray crystallography

## Abstract

The BEN domain-containing transcription factors regulate transcription by recruiting chromatin-modifying factors to specific chromatin regions *via* their DNA-binding BEN domains. The BEN domain of BANP has been shown to bind to a CGCG DNA sequence or an AAA-containing matrix attachment regions DNA sequence. Consistent with these *in vivo* observations, we identified an optimal DNA-binding sequence of AAATCTCG by protein binding microarray, which was also confirmed by our isothermal titration calorimetry and mutagenesis results. We then determined crystal structures of the BANP BEN domain in apo form and in complex with a CGCG-containing DNA, respectively, which revealed that the BANP BEN domain mainly used the electrostatic interactions to bind DNA with some base-specific interactions with the TC motifs. Our isothermal titration calorimetry results also showed that BANP bound to unmethylated and methylated DNAs with comparable binding affinities. Our complex structure of BANP-mCGCG revealed that the BANP BEN domain bound to the unmethylated and methylated DNAs in a similar mode and cytosine methylation did not get involved in binding, which is also consistent with our observations from the complex structures of the BEND6 BEN domain with the CGCG or CGmCG DNAs. Taken together, our results further elucidate the elements important for DNA recognition and transcriptional regulation by the BANP BEN domain-containing transcription factor.

The BEN (BANP, E5R, and NAC1) domain is a ∼100-residue protein domain found in metazoans and viruses, but missing in nematodes and urochordates (1). There are nine BEN domain–containing transcription factors in the human genome, *i.e.*, BANP, BEND2-7, and NAC1/2 (Fig. 1*A*). BANP (BTG3-associated nuclear protein, also named BEND1 or SMAR1 (scaffold/matrix-associated region-binding protein 1)), BEND4/5/6/7, and NAC1/2 contain a single BEN domain, while BEND2/3 contain multiple BEN domains (Fig. 1*A*) (2, 3). Most of these BEN domain-containing transcription factors have been reported to function in recruiting chromatin-modifying factors to assemble higher-order chromatin structure in transcriptional regulation (1).

**Figure 1:**
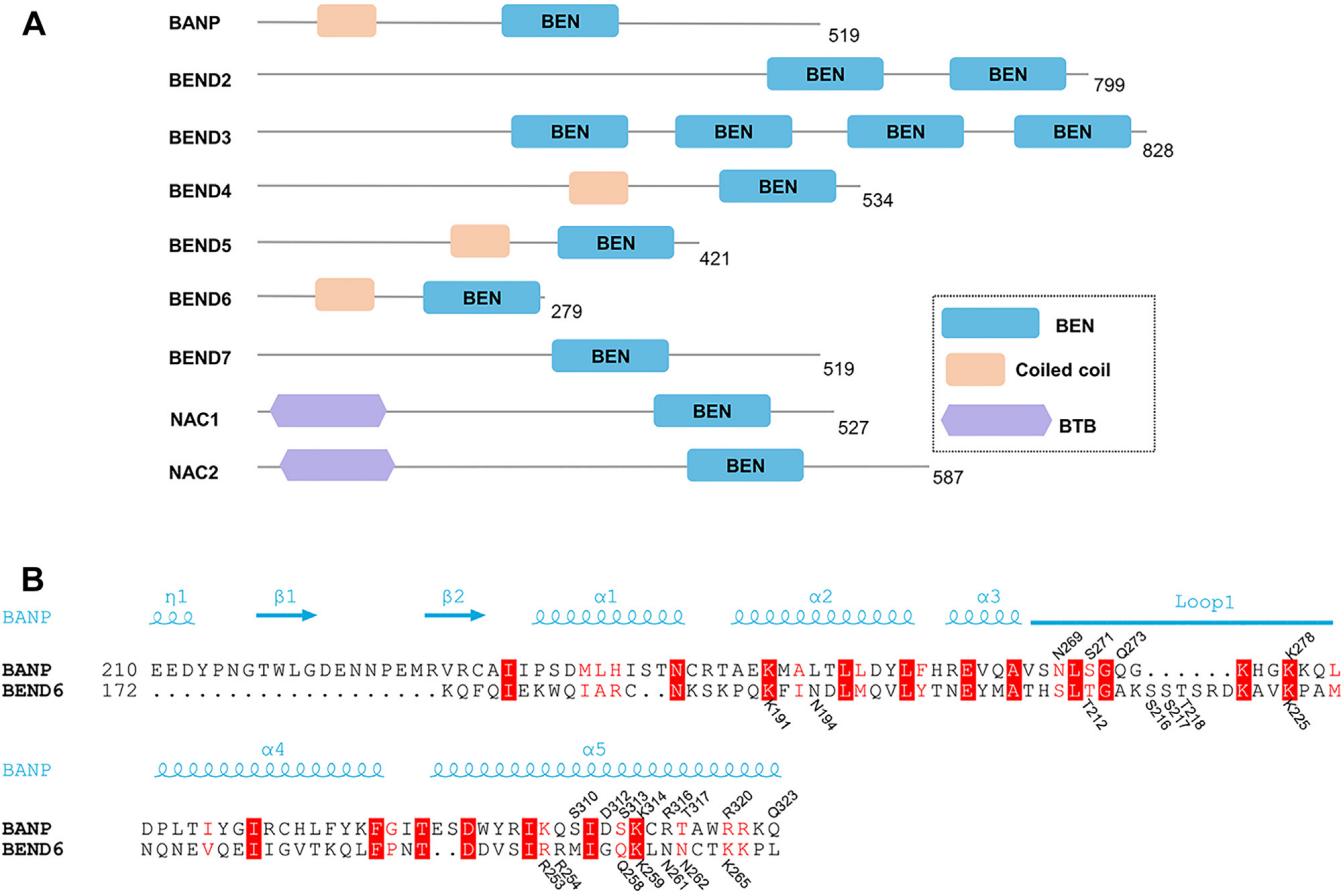
**Domain structure and sequence alignment of the BEN domain proteins**. *A*, domain organization of human BEN domain proteins: BANP (NP_001167014.1), BEND2 (NP_699177.2), BEND3 (NP_001073919.1), BEND4 (NP_997289.2), BEND5 (NP_078879.2), BEND6 (NP_689944.2), BEND7 (XP_011517694.1), NAC1 (NP_443108.1), NAC2 (NP_653254.1). The protein length is marked at the right of each protein. *B*, sequence alignment of the BEN domains of BANP and BEND6. The sequences were analyzed using the multiple sequence and structure alignment server PROMALS3D based on the BANP BEN domain structure. The DNA-interacting residues of the BANP and BEND6 BEN domains are marked at the *top* and *bottom* of their sequences, respectively. BEN, BANP, E5R, and NAC1; BANP, BTG3-associated nuclear protein; BTB: Broad-Complex, Tramtrack and Bric a brac.

Both human and mouse BANPs have been shown to activate some essential metabolic genes in pluripotent stem cells and terminally differentiate neuronal cells by recognizing a consensus DNA sequence motif of CGCG located in the transcription start sites of these genes (4). Zebrafish BANP regulates genes involved in DNA replication and chromosome segregation during embryonic development, and the CGCG motif sequence was also identified in some of their target genes (5). BEND2 binds to GA-rich DNA motifs and functions as a key regulator of transcriptional repression in mouse spermatogenesis (6). BEND3 modulates chromatin structure through diverse mechanisms (7): it could tether nucleolar remodeling complex at the rDNA promoters and repress transcription of the rDNA genes (8) or regulate transcriptional repression by interacting with transcription factor SALL4 and histone deacetylase 1, two key components of the nucleosome remodeling and deacetylase complex (9). BEND3 has also been shown to be a CpG island binder binding a DNA sequence motif of CCCACGCG and either stabilizes the polycomb repressive complex 2 at bivalent genes in embryonic stem cells, which prevents premature activation of CpG island-containing bivalent genes during differentiation (10), or recruits polycomb repressive complex 2 to major satellites and modulates their switch from the constitutive heterochromatin structure to the facultative one (11). BEND4, together with BEND5, interacts with pluripotency transcription factors, such as OCT4, KLF4, and SOX2, and functions as chromatin boundary factors to facilitate transcriptional activation during germ cell differentiation (12). BEND6, as a CSL (CBF1, Suppressor of Hairless, Lag-1) corepressor, directly interacts with the Notch transcription factor CBF1 and inhibits neural stem cell self-renewal (13). NAC1 and NAC2 share ∼85% sequence similarity. Although different DNA sequence preferences have been demonstrated for NAC1 (14) and NAC2 (15), recently it was shown that the BEN domains of NAC1 and NAC2 exhibit the same binding preference for the ACATGT motif (16, 17). NAC1 regulates embryonic stem cell differentiation by coordinating with pluripotency factors including OCT4, TCF3, and SOX2 (14) and could also interact with CoREST and HDAC 3/4 for transcriptional repression (2, 3). NAC2 recruits the nucleosome remodeling and deacetylase complex to the internal promoter of the E3 ligase HDM2 to repress the expression of HDM2 (18). To date, the function of BEND7 is still poorly characterized.

By ChIP-seq analysis of the genomic DNA binding sequence, BANP was found to bind to a consensus sequence of CGCG within the TCTCGCGAGA sequence context (4). Interestingly, BANP was initially identified as a matrix-associated protein involved in gene repression by binding to the matrix attachment regions (MARs) DNA (19, 20, 21). For example, BANP recognizes the hexanucleotide CAAAGA-containing MARs sequence at the 5′ long terminal repeat of HIV-1, suppressing the HIV-1 replication and virion production (20). Similarly, BANP suppresses the oncogene E6 by recruiting the histone deacetylase 1 corepressor complex to the MARs element of the E6 promoter in HPV18-infected cervical adenocarcinoma cells (21). BANP also mediates the suppression of apoptotic genes BAX and PUMA through binding to their MARs elements during mild DNA damage, leading to cell cycle arrest (19). These findings suggest that BANP could bind to diverse DNA motifs, acting as either a transcription activator or a transcription repressor.

Nevertheless, how BANP recognizes its target DNA to get engaged in transcriptional regulation remains to be elucidated. In this study, we tried to understand the DNA binding mechanism of the BANP BEN domain by means of structural, biophysical, and biochemical analyses. By using the protein binding microarray (PBM) assay, we identified an *in vitro* optimal DNA binding sequence of AAATCTCG, which was also confirmed by our isothermal titration calorimetry (ITC) and mutagenesis results. Our ITC results also showed that BANP was able to bind any DNA sequence, albeit ∼15-fold weaker. Consistently, our structural studies revealed that the BEN domain of BANP mainly used the electrostatic interactions to bind DNA with some base-specific interactions with the TC motifs. We also solved the structure of the BANP BEN domain in complex with a mCGCG DNA, as well as the structures of the BEND6 BEN domain in complex with the CGCG and CGmCG DNAs, respectively, which revealed that these two BEN domains bound to the unmethylated and the methylated DNAs in a similar mode.

## Results and discussion

### Structural basis of DNA recognition by the BEN domain of BANP

**Figure 2:**
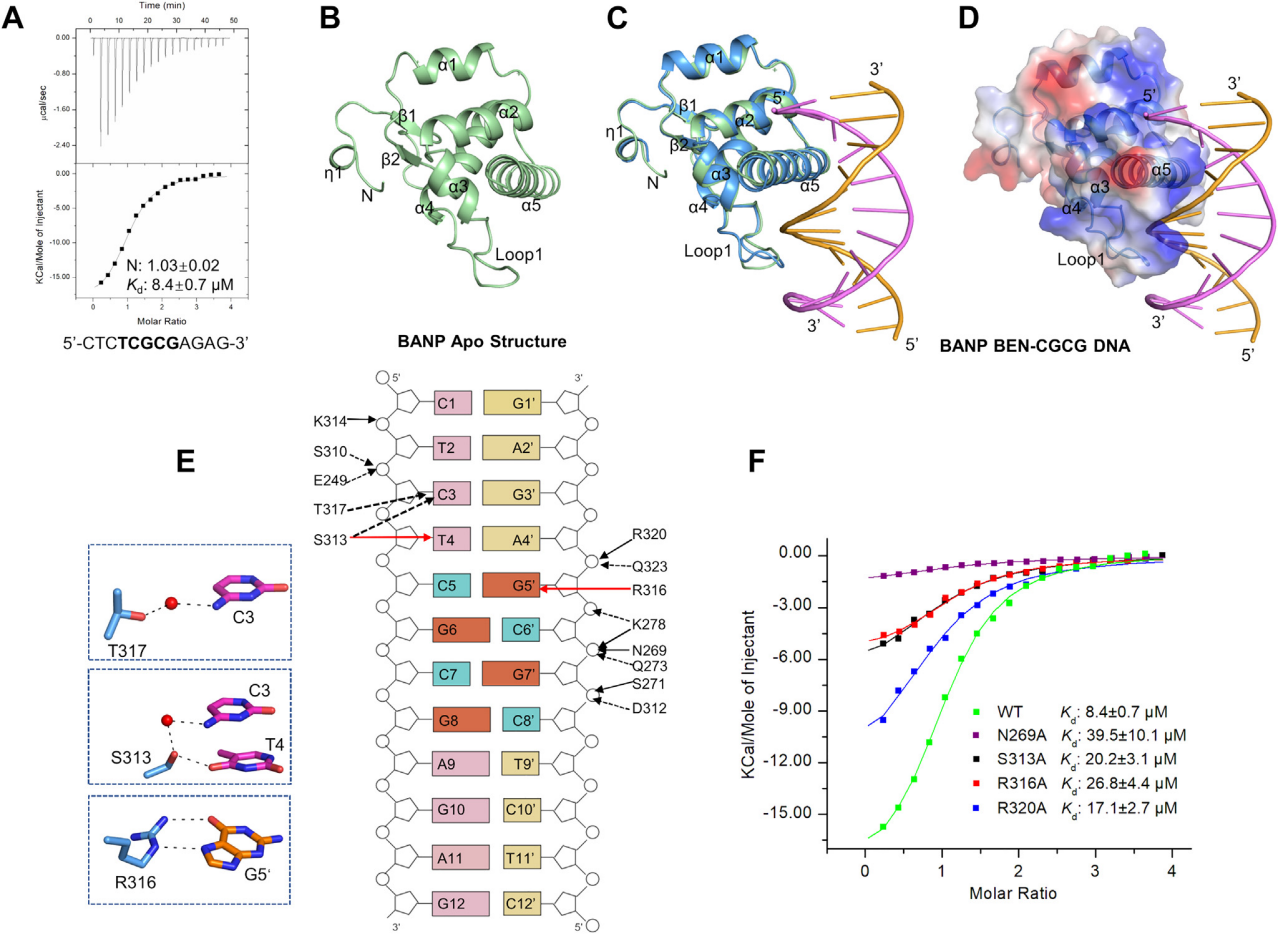
**Structural basis for the recognition of a CGCG motif containing DNA by the BEN domain of BANP**. *A*, ITC binding curve of the BANP BEN domain to the CGCG motif DNA. The palindromic DNA sequence is 5’-CTCTCGCGAGAG-3’. The error is a fitting error of the ITC titration curve. *B*, structure of the BANP BEN domain in apo form. *C*, superposition of the structures of the BANP BEN domain in apo form (*green*) and in complex with the DNA (*blue*). *D*, electrostatic surface potential of the BANP BEN domain in complex with the CGCG DNA viewed in the same orientation as (*C*). Electrostatic surface potential was generated by PyMOL. *E*, schematic diagram showing the interactions between the BANP BEN domain and the CGCG motif DNA. The cytosines and guanines of the CGCG motif are colored in *cyan* and *orange*, respectively. The direct base interactions are marked as *red solid arrows*, the direct phosphate group interactions are marked as *black solid arrows*, and the water-mediated hydrogen bonds are indicated by *black dashed arrows*. Detailed base interactions are also shown in the *blue zoom-in boxes. F*, ITC binding results of the BANP BEN domain mutants to the CGCG motif DNA. The errors are fitting errors of the ITC titration curves. BANP, BTG3-associated nuclear protein; BEN, BANP, E5R, and NAC1; ITC, isothermal titration calorimetry.

To explore how the BEN domain of BANP recognizes DNA, we tried to determine its complex structure with DNA. The BEN domain of BANP has been reported to recognize a consensus sequence of CGCG within the TCTCGCGAGA sequence context (4). We then synthesized a 12-mer DNA fragment with a palindromic sequence of CTCTCGCGAGAG for the crystallization study. We first measured its binding ability to the human BANP BEN domain by ITC, which showed that the BEN domain of BANP bound to the palindromic 12-mer DNA with a *K*_d_ of 8.4 μM (Fig. 2*A*). We then carried out crystallization experiments and solved the structures of the BANP BEN domain in the apo form and in complex with the palindromic DNA by using the short and long constructs of the human BEN domain (aa 208-324 and aa 208-347), respectively (Table S1). Structural analysis showed that the BEN domain of BANP mainly consisted of a short N-terminal β-hairpin, which was packed against a helix-bundle core of 5 α-helices (Fig. 2, *B* and *C*). In the BANP-DNA complex structure, the extra C-terminal region after the helix α5 in the longer construct is disordered and unresolved in the structure. The structures of the BANP BEN domain in the apo form and the DNA complex overlaid very well with an RMSD of 0.68 Å over its 101 Cα atoms, suggesting that the DNA binding did not cause significant conformational changes (Fig. 2*C*).

In the DNA-bound structure, the DNA binding surface of the BANP BEN domain was largely positively charged, which conferred electrostatic interactions with the DNA (Fig. 2*D*). The BANP BEN domain interacted with the palindromic DNA mainly through its C-terminal α5 helix, which inserted into the major groove of DNA (Fig. 2*C*). Specifically, the side chain of S313 from the α5 helix formed a hydrogen bond with the base of T4, and the side chain of R316 formed two hydrogen bonds with the base of G5′ that is the second G of the CGCG motif (Fig. 2*E*). S313 and T317 also formed two water-mediated hydrogen bonds with the base of C3 (Fig. 2*E*). In addition to these base-engaged interactions, S310, D312, K314, R320, and Q323 from the α5 helix and N269, S271, Q273, and K278 from the loop connecting α3 and α4 (called Loop 1 thereafter) formed several hydrogen bonds or salt bridges with the backbone of DNA (Fig. 2*E*). To explore the importance of these residues of BANP in DNA binding, we performed mutagenesis and ITC binding assays. The results showed that mutating N269, S313, R316, and R320 of BANP to alanine individually reduced its DNA binding affinity by ∼2-5-fold compared to the WT BANP (Figs. 2*F* and S2*A*). Taken together, our structural and biochemical data demonstrate that the BANP BEN domain mainly utilized the electrostatic interactions to bind DNA with some base-specific interactions with the TC motifs by the α5 helix and Loop 1 of BANP.

### The BANP BEN domain preferentially bound to a consensus sequence of AAATCTCG

**Figure 3:**
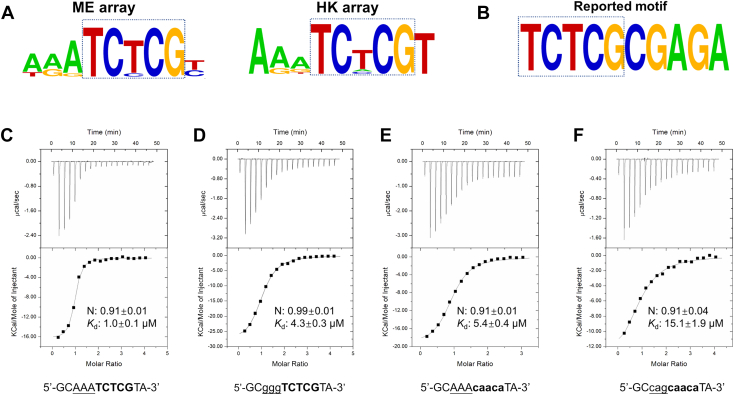
**DNA sequence-specificity analysis of the BANP BEN domain by PBM and ITCbinding assays**. *A*, predominant motifs from the ME and HK PBM assays, respectively. Predominant motifs were generated using the PWM_align algorithm. *B*, the modified reported BANP binding motif. *C*, ITC binding curve of the BANP BEN domain with the DNA sequence from PBM data. *D*–*F*, ITC binding curves of the BANP BEN domain with different DNA mutants. Only one strand of the DNA duplex is shown. The errors are fitting errors of the ITC titration curves. BANP, BTG3-associated nuclear protein; BEN, BANP, E5R, and NAC1; ITC, isothermal titration calorimetry; PBM, protein-binding microarray; PWM, position weight matrix.

Our DNA-bound structure of the BANP BEN domain showed that the BANP BEN domain specifically recognized the bases of the T4C5 dinucleotide in the CTCTCGCGAGAG sequence. Other than that, it made few base-specific interactions with the DNA sequence (Fig. 2*E*). To further investigate the optimal DNA binding sequence of the BANP BEN domain, we used the PBM to find out its consensus binding motif *in vitro*. In the PBM experiments, two kinds of arrays, named ME and HK, which guaranteed an unbiased estimate of the DNA binding sequence preference of the target proteins of interest, were constructed (22). Our PBM results revealed that the BANP BEN domain preferred a DNA motif of TCXCG (X= T>> A∼C) with the highest scoring sequence AAATCTCGT from both the ME and HK arrays (Figs. 3*A* and S3). Notably, aligned with the reported BANP binding motif TCTCGCGAGA derived from the ChIP-seq analysis (4), we found that the consensus sequence TCTCG and its complementary sequence CGAGA from the PBM array analysis match the 5′- and 3′-end sequences of the reported BANP binding motif TCTCGCGAGA, respectively (Fig. 3, *A* and *B*) (4). In addition to the TCTCG motif, the AAA moiety from the PBM data reminded us of the binding of BANP to the CAAAGA-containing MARs DNA sequence in the HIV-1 long terminal repeats (20). Mutating the three As to Gs has been reported to reduce the binding of BANP to the HIV-1 MARs DNA (20). Taken together, our PBM data identified the optimal binding sequence of the BANP BEN domain as AAATCTCG, whose AAA and TCTCG moieties have been identified in the genomic binding sites of BANP in pluripotent stem cells (4), and the viral DNA binding site by the host transcription factor BANP in HIV transcription *in vivo* (20).

To further confirm this optimal binding motif, we synthesized a 12-mer DNA fragment of the sequence GCAAATCTCGTA based on the highest scoring sequence AAATCTCGT from our PBM data and measured its binding affinity to the BANP BEN domain by ITC. Our ITC result showed that the BANP BEN domain bound to the GCAAATCTCGTA sequence with a *K*_d_ of 1.0 μM, which is more than 8-fold stronger than the CGCG-containing motif (Fig. 3*C* and Table 1). When we mutated the AAA motif to cag or ggg, its binding affinity was reduced by ∼3-4-fold (Figs. 3*D* and S1 and Table 1). When we mutated the TCTCG motif to caaca, its binding affinity was reduced by more than 5-fold (Fig. 3*E* and Table 1). However, when we replaced the TCTCG motif with the TCGCG motif, the motif identified from the ChIP-seq data (4), its binding affinity (*K*_d_ of 1.5 μM) is just slightly weaker than the optimal AAATCTCG motif (Table 1 and Fig. S1). Furthermore, we synthesized some 12-mer DNA sequences containing neither motif and found that they could still bind to BANP reasonably well with binding affinities of ∼15 μM, which explains our structural observations that the BANP BEN domain mainly used electrostatic interactions to bind DNA with some base-specific interactions with the TC nucleotides (Figs. 3*F* and S1 and Table S1).

**Table 1:** Binding affinities of the BEN domain of BANP to different DNA sequences

DNA sequences	N	*K*_d_ (μM）	ΔH (kcal·mol^−1^)	-T·ΔS (kcal·mol^−1^)	ΔG (kcal·mol^−1^)
5’-CTC**TCGCG**AGAG-3’^a^	1.03 ± 0.02	8.4 ± 0.7	−19.2 ± 0.5	12.3	−6.9
5’-GCAAA**TCTCG**TA-3’^b^	0.91 ± 0.01	1.0 ± 0.1	−16.4 ± 0.2	8.2	−8.2
5’-GCcag**TCTCG**TA-3’	1.02 ± 0.01	2.9 ± 0.3	−21.9 ± 0.3	14.3	−7.6
5’-GCggg**TCTCG**TA-3’	0.99 ± 0.01	4.3 ± 0.3	−29.0 ± 0.4	21.7	−7.3
5’-GCAAA**caaca**TA-3’	0.91 ± 0.01	5.4 ± 0.4	−19.80 ± 0.3	12.6	−7.2
5’-GCAAA**TCGCG**TA-3’	0.97 ± 0.01	1.5 ± 0.2	−24.7 ± 0.5	16.7	−8.0
5’-GCcag**caaca**TA-3’	0.91 ± 0.04	15.1 ± 1.9	−15.1 ± 1.0	8.5	−6.6
5’-CTC**tggcg**AGAG-3’	0.99 ± 0.04	14.8 ± 2.3	−6.0 ± 0.4	−0.6	−6.6
5’-CTC**acgcg**AGAG-3’	1.00 ± 0.05	14.3 ± 2.6	−3.6 ± 0.2	−3.0	−6.6
5’-CTC**gagcg**AGAG-3’	0.93 ± 0.05	14.1 ± 3.0	−6.4 ± 0.5	−0.2	−6.6
5’-CTC**TmCGCG**AGAG-3’	0.90 ± 0.03	17.8 ± 1.5	−19.4 ± 0.9	12.9	−6.5
5’-CTC**TCGmCG**AGAG-3’	0.98 ± 0.02	7.6 ± 0.3	−35.2 ± 0.7	28.2	−7.0

a Reported DNA-binding motif of BANP in literature, which is also used for crystallization in this study.

b DNA-binding motif of BANP identified by protein binding microarray in this study. Only one strand of the DNA duplex is shown in the table. The mutated nucleotides are shown in lower case. The TCTCG and TCGCG motifs are shown in bold, and the AAA motif found in the matrix attachment regions (MARs) DNA sequence is underlined. The errors are fitting errors of the ITC titration curves.

In order to understand why the AAA motif could enhance the DNA binding to BANP, we tried to determine the complex structure of the BANP BEN domain with the GCAAATCTCGTA DNA to no avail. However, the A-tract structure has been reported to introduce DNA bending and facilitate high-affinity sequence-specific protein-DNA interactions (23), which warrant future studies.

### Structural basis for the methylation-insensitive DNA binding by the BEN domain

**Figure 4:**
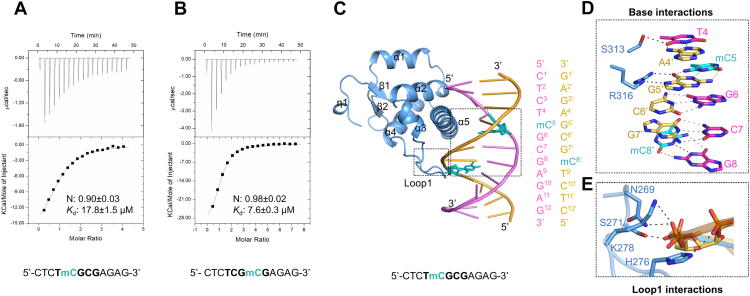
**Crystal structure of the BANP BEN domain in complex with mCGCG DNA**. *A* and *B*, ITC binding curves of the BANP BEN domain to different methylated DNA. Only one strand of the DNA duplex is shown. The errors are fitting errors of the ITC titration curves. *C*, overall structure of the BANP BEN domain in complex with the mCGCG DNA. *D*, the base interactions of BANP BEN domain with methylated DNA. The interacting residues and bases are shown in *stick* models. *E*, the interactions of Loop 1 with the phosphate group of DNA. Hydrogen bonds formed between protein and DNA are marked as *black dashed lines*, while hydrogen bonds of DNA bases are marked as *gray dashed lines*. BANP, BTG3-associated nuclear protein; BEN, BANP, E5R, and NAC1; ITC, isothermal titration calorimetry.

BANP has been reported to prefer unmethylated DNA, and methylation in the CGCG motif significantly reduces its binding affinity to BANP (4). Here, we measured the binding abilities of the BANP BEN domain to DNAs containing mCGCG and CGmCG, respectively. Our ITC results showed that the mCGCG DNA displayed only a 2-fold weaker binding affinity to the BANP BEN domain than the unmethylated CGCG DNA, and the CGmCG DNA exhibited a similar binding affinity to the unmethylated CGCG DNA (Figs. 4, *A* and *B* and S4 and Table 1). Hence, our binding assay results indicated that the BANP BEN domain was likely a methylation-insensitive DNA binder.

To illustrate the molecular mechanism of the BANP BEN domain in recognizing the methylated DNA, we obtained the crystal structure of the BANP BEN domain bound to an mCGCG DNA (Table S1). Similarly, the C-terminal region after the helix α5 is disordered in the BANP-mCGCG structure. Superposition of the structures of the BANP-CGCG and BANP-mCGCG complexes revealed that these two structures superimposed well with an RMSD of 0.34 Å over their 104 Cα atoms, suggesting that the methylation of the cytosine within the DNA did not cause significant conformational changes. Like the BANP-CGCG structure, the BANP BEN domain interacted with the methylated DNA mainly through its α5 helix (Fig. 4*C*). Similarly, in the BANP-mCGCG complex, the side chain of S313 formed a hydrogen bond with the base of T4, and the side chain of R316 formed two hydrogen bonds with the base of G5′ that is complementary with the methylcytosine, while the methyl group of mC was not involved in the BANP binding (Fig. 4*D*). In addition to the base interactions, Loop 1 also was involved in the interactions with the backbone of DNA *via* the residues N269, S271, H276, and K278 (Fig. 4*E*).

**Figure 5:**
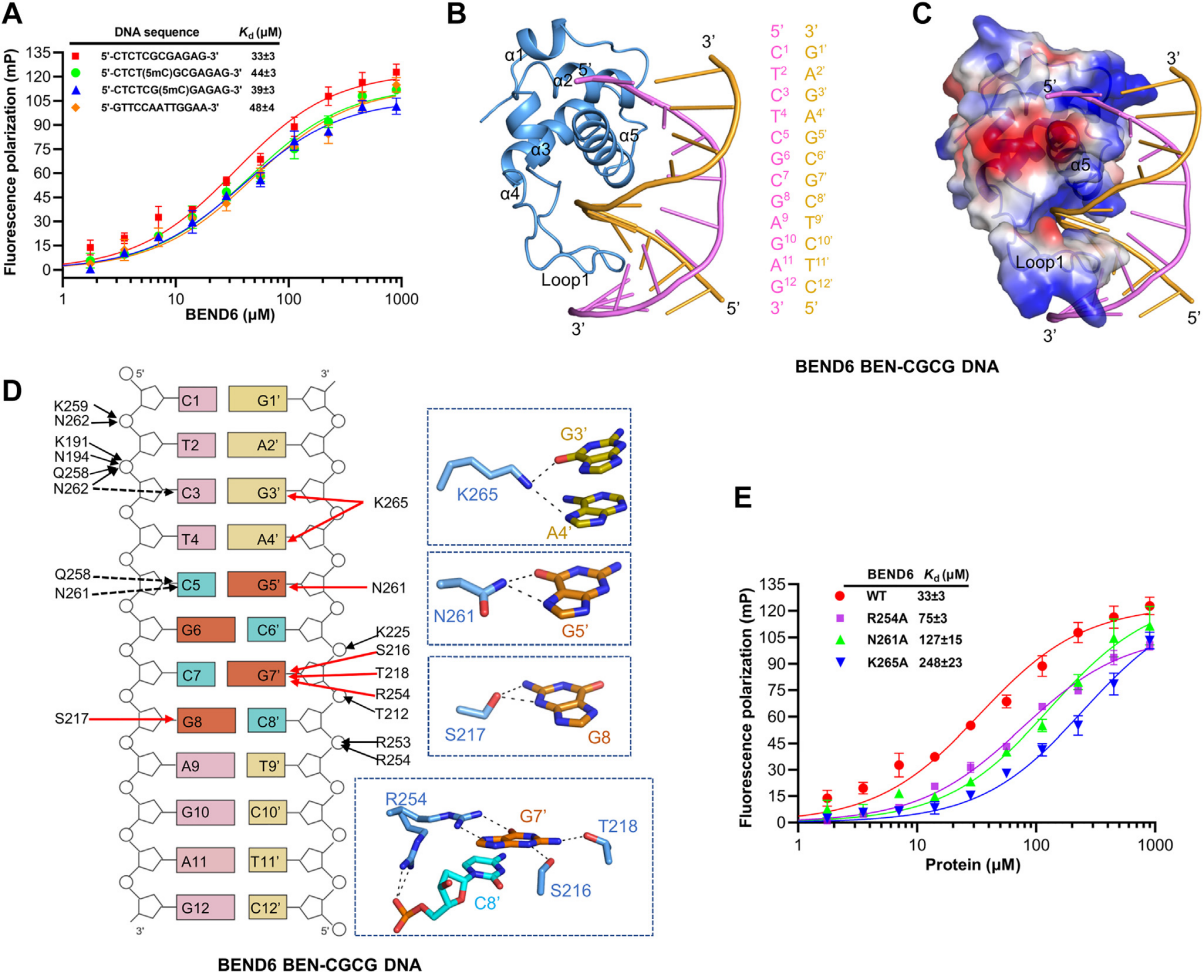
**Crystal structure of the BEND6 BEN domain in complex with a CGCG motif containing DNA**. *A*, FP binding assay results of the BEND6 BEN domain to different DNA sequences. Only one strand of the DNA duplex is shown. The errors for *K*_d_ values are the fitting errors, and the experimental errors are based on the average value of the replicates. *B*, overall structure of the BEND6 BEN domain in complex with the CGCG motif DNA. The palindromic DNA sequence is 5’-CTCTCGCGAGAG-3’. *C*, electrostatic surface potential of the BEND6 BEN domain in complex with the CGCG motif DNA viewed in the same orientation as (*B*). Electrostatic surface potential was generated by PyMOL. *D*, schematic diagram showing the interactions between the BEND6 BEN domain and the CGCG DNA. The cytosines and guanines of the CGCG motif are colored in *cyan* and *orange*, respectively. The direct base interactions are marked as *red solid arrows*, the direct phosphate group interactions are marked as *black solid arrows*, and the water-mediated hydrogen bonds are indicated by *black dashed arrows*, respectively. Detailed base interactions are also shown in the *blue zoom-in boxes*. *E*, FP binding assay results of the BEND6 BEN domain mutants to the CGCG DNA. The errors for *K*_d_ values are the fitting errors, and the experimental errors are based on the average value of the replicates. FP, fluorescence polarization.

To further investigate the role of cytosine methylation in BEN domain binding, we successfully obtained the structures of the BEND6 BEN domain in complexes with the CGCG motif and CGmCG motif containing DNAs, respectively (Table S1). Our binding results also revealed that the BEN domain of BEND6 exhibited comparable binding affinities to the methylated and unmethylated CGCG motif DNA sequences with *K*_d_ of ∼39 μM and ∼33 μM, respectively (Fig. 5*A*). In both structures, the last six amino acid residues (K266 to K271) after the helix α5 are disordered. Structural analysis revealed that the BEND6 BEN domain adopted a canonical α-helical architecture but lacked the N-terminal β-hairpin compared to that of the BANP BEN domain (Figs. 2*B* and 5*B*). Like the BANP BEN domain as well as other reported BEN domains (10, 15, 17, 24), the α5 helix of BEND6 inserted into the major groove of DNA, while a longer Loop 1 stepped into the minor groove of DNA, which together contributed to the electrostatic interactions with the DNA (Fig. 5, *B* and *C*).

In the BEND6-CGCG DNA structure, the side chain of K265 from the α5 helix formed two hydrogen bonds with the bases of G3′ and A4′, respectively, while the side chain of N261 from the α5 helix made hydrogen-bonding interactions with the base of G5′. The side chains of R254 from the α5 helix and S216, T218 from the Loop 1 formed four hydrogen bonds with the base of G7′. In addition, the side chain of S217 also made two hydrogen bonds with the base of G8 (Fig. 5*D*). Moreover, the side chains of Q258, N261, and N262 formed water-mediated hydrogen bonds with the bases of C3 and C5, respectively (Fig. 5*D*). In addition to these base interactions, the side chains of K191, N194, T212, K225, R253, Q258, K259, and N262 formed several hydrogen bonds and salt bridges with the DNA backbone (Fig. 5*D*). Interestingly, the side chain of R254 adopted two conformations, one of which made interactions with the base as mentioned above and the other made interactions with the DNA backbone (Fig. 5*D*). The importance of the base-interacting residues was further examined by mutagenesis and binding assay with fluorescence polarization (FP). The results showed that mutating R254, N261, and K265 to alanine individually reduced the binding of the BEND6 BEN domain with the CGCG DNA by ∼2-6-fold, suggesting that these residues played a role in DNA binding (Figs. 5, *A* and *E*, and S2*B*).

**Figure 6:**
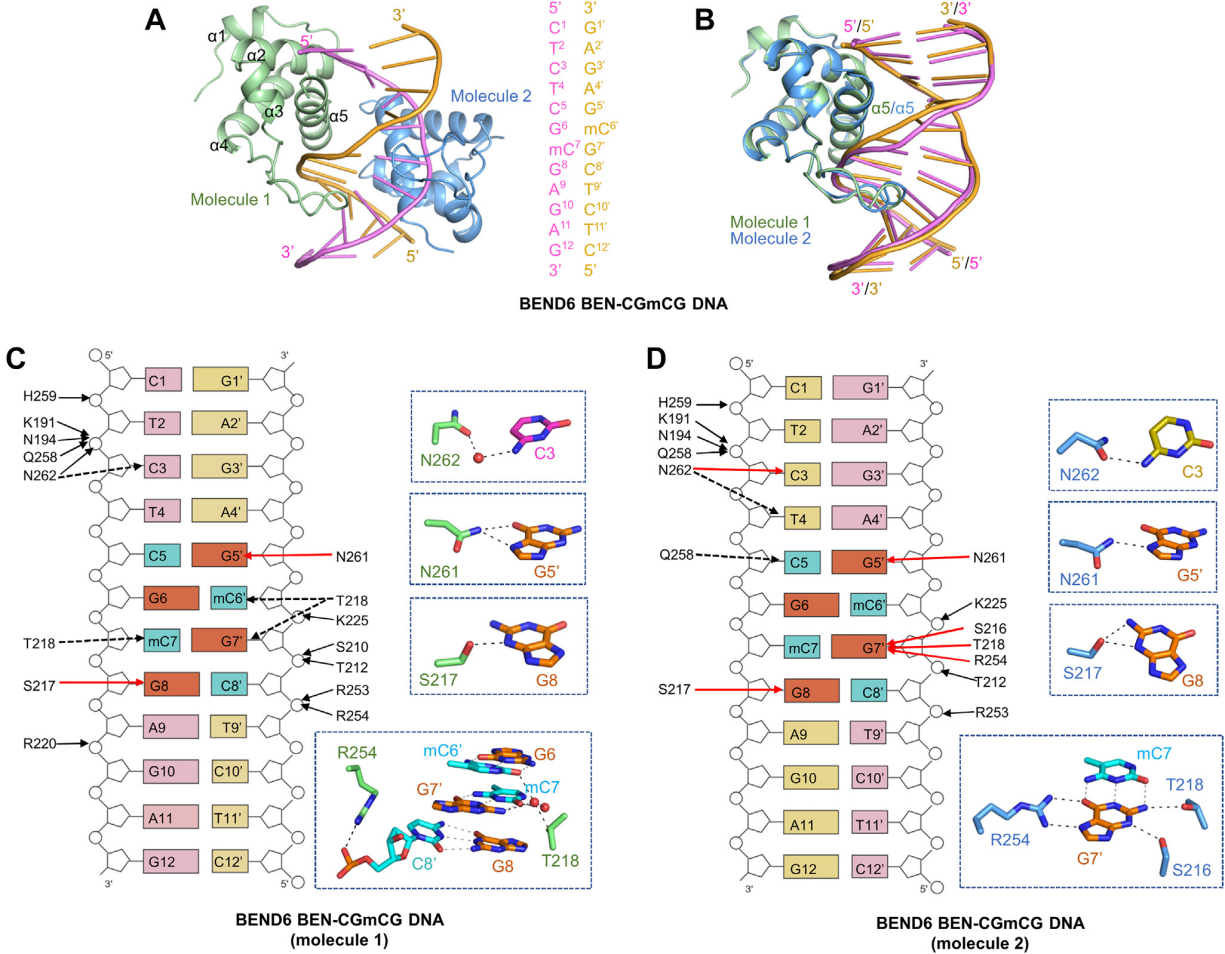
**Crystal structure of the BEND6 BEN domain in complex with CGmCG DNA**. *A*, overall structure of the BEND6 BEN domain in complex with a CGmCG DNA. *B*, superposition of the structures of the two CGmCG DNA-bound BEND6 BEN molecules. *C* and *D*, schematic diagrams showing the interactions of the two BEND6 BEN molecules with the CGmCG DNA. The cytosines (methylcytosines) and guanines of the CGmCG motif are colored in *cyan* and *orange*, respectively. The direct base interactions are marked as *red solid arrows*, the direct phosphate group interactions are marked as *black solid arrows*, and the water-mediated hydrogen bonds are indicated by *black dashed arrows*, respectively. Detailed base interactions are also shown in the *blue dotted boxes*.

In the BEND6-CGmCG structure, two BEND6 BEN domain molecules bound to a DNA duplex in an asymmetric unit (Fig. 6*A*). The two BEND6 molecules could be superimposed very well (Fig. 6*B*), and they bound to the DNA in roughly the same binding mode (Fig. 6, *C* and *D*). For instance, both BEND6 molecules used N262, N261, and S217 to make hydrogen-bonding interactions with the bases of C3, G5’, and G8 of the DNA, respectively, although N262 of the BEND6 molecule 1 formed a water-mediated interaction with the base of C3 (Fig. 6*C*), while N262 of the BEND6 molecule 2 formed a direct interaction with the base of C3 (Fig. 6*D*). Moreover, we found that the binding mode of the CGmCG DNA to the BEND6 BEN domain was very similar to that observed in the BEND6-CGCG DNA complex (Figs. 5 and 6). Similar to the BEND6-CGCG DNA structure, R254 interacted with the base of G7’ in one molecule (Fig. 6*D*) and interacted with the DNA backbone in the other molecule (Fig. 6*C*). Taken together, our structural data demonstrated that the BANP and BEND6 BEN domains bound to the unmethylated and methylated DNA in a similar mode, consistent with our binding data that the BANP and BEND6 BEN domain were methylation-insensitive DNA binders.

### Structural comparisons of DNA recognition by BEN domains

**Figure 7:**
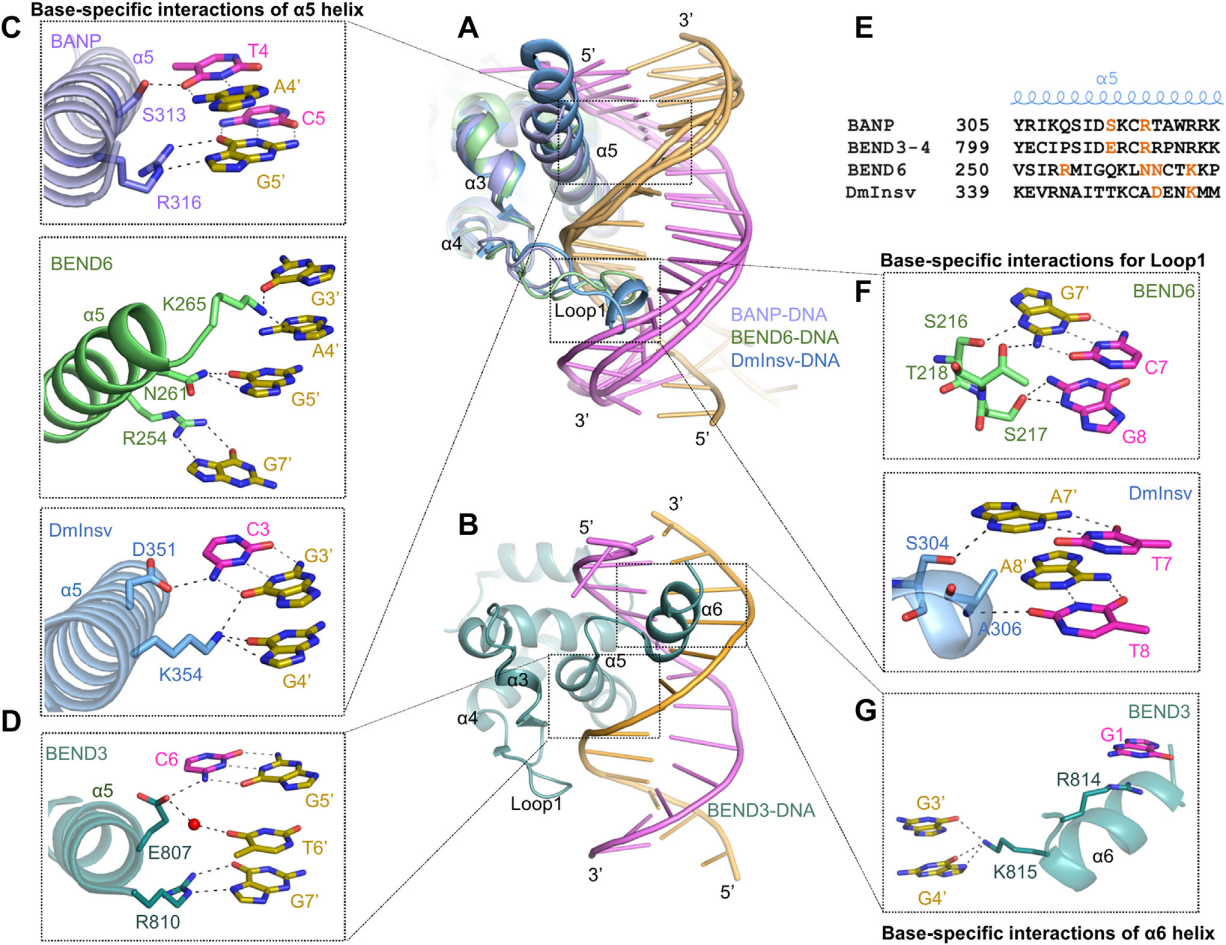
**Structural comparisons of different DNA-bound BEN domains**. *A*, superposition of the structures of the BANP (*purple*)-DNA, BEND6 (*green*)-DNA and DmInsv (PDB: 4IX7, *blue*)-DNA complexes. *B*, structure of human BEND3 BEN4 domain in complex with DNA (PDB: 7W27, *green*). *C* and *D*, the base interactions of the α5 helix with the DNA in the BANP, BEND6, DmInsv, and BEND3 structures, respectively. *E*, sequence alignment of the α5 helices from the BEN domains of BANP, BEND6, DmInsv, and BEND3. The direct base-interacting residues are colored in *orange*. *F*, the base interactions of the Loop 1 with the DNA in the BEND6 and DmInsv structures, respectively. *G*, the base interactions of the α6 helix with the DNA in the BEND3 BEN4 structure. The interacting residues and bases are shown in *stick models*. Hydrogen bonds formed between protein and DNA are marked as *black dashed lines*, while hydrogen bonds of DNA bases are marked as *gray dashed lines*.

The structures of the DNA-bound mammalian BEND3 BEN4 domain and two Drosophila BEN-solo factors have been determined (10, 15, 17, 24). Structural comparison showed that all of these BEN domains use a positively charged surface to recognize and bind the DNA, which is very similar to that seen in the structures of the BEN domains of BANP and BEND6 in complexes with both unmethylated and methylated DNAs reported in this study (Figs. 2*D* and 5*C* and S5). Further structural analysis revealed that although all these BEN domains bind their target DNAs mainly through the insertion of the C-terminal α5 helix into the major groove of DNA, the residues of the α5 helices involved in the base interactions vary from one to another (Fig. 7, *A*–*E*), explaining why they exhibit distinct DNA sequence preference (10, 15, 17, 24). In addition to the α5 helix, the BEN domains of BEND6 and DmInsv also contained a relatively long Loop 1, which inserts into the minor groove of DNA to form additional base-specific interactions with the DNA (Fig. 7*F*) (15, 24). The equivalent Loop 1 in the BEN domain of BANP was shorter and just interacted with the backbone phosphate groups of the DNA (Fig. 7*A*). Although the BEND3 BEN4 domain also harbors a shorter Loop 1, it contains an extra helix α6 following the α5 helix, which inserts into the major groove of DNA and provides additional base-specific interactions with the DNA (Fig. 7, *B* and *G*) (10, 17). Therefore, the different structural characteristics of individual BEN domains explain why they display distinct DNA binding preferences and binding modes.

In conclusion, our structural, biophysical, and biochemical studies of the BANP BEN domain demonstrated its DNA recognition preference and mechanism, which also expanded our understanding of the variety of selectivity and binding modes of human BEN domains.

## Experimental procedures

### Protein expression and purification

The DNA fragments encoding the human BANP BEN domain (aa 208-347 and aa 208-324) and the BEND6 BEN domain (aa 170-271) were subcloned into the pET28-SUMO and pET28-MHL vectors, which attach an N-terminal His_6_-SUMO tag and His_6_-tag followed by a tobacco etch virus cleavage site, respectively. All recombinant proteins were overexpressed in the *Escherichia coli* BL21 (DE3) induced with 0.5 mM IPTG at 14 °C. Subsequently, the bacterial cells were collected and resuspended in a lysis buffer consisting of 500 mM NaCl, 25 mM Tris-HCl (pH 7.5), 5% glycerol, and 5 mM imidazole. Following centrifugation at 16,000 g for 60 min, the supernatants of the target proteins were purified using Ni-NTA resin (Qiagen). Then the tobacco etch virus protease was used to remove the His_6_-SUMO or His_6_-tag from the recombinant BEN domain proteins. The target proteins were further purified using a Superdex 75 gel filtration column (GE HealthCare) with a buffer containing 150 mM NaCl and 20 mM Tris-HCl (pH 7.5) with or without DTT. The BANP and BEND6 mutants were generated by the QuickChange site-directed mutagenesis (Agilent Technologies) using the BANP (aa 208-347) and BEND6 (aa 170-271) expression constructs as the template, respectively. Mutant proteins were expressed and purified as described for the WT proteins.

### ITC assay

All DNA oligos used in this study were purchased from General Biosystems Co. Ltd and then annealed to DNA duplex by a PCR instrument in the same buffer as for the proteins. The concentrations of proteins and DNA samples used for ITC binding assays were from 50 to 90 μM and 1 mM, respectively. All the ITC assays were performed at 25 °C using MicroCal ITC200 (Malvern). BANP protein was added to the sample cell, while DNA was filled into syringe. Each titration consisted of 19 injections, in which the first was set as 0.4 μL and the rest was set as 2 μL. Three replicates were carried out for the binding of BANP with the DNA sequence AAATCTCG and one of its mutants, and methylated and unmethylated CGCG DNA, which showed that all the ITC data were reproducible. For most of the other ITC experiments of BANP with different DNA sequences, we experimented just once. The stoichiometry (N), dissociation constant (*K*_d_), enthalpy (ΔH), entropy (ΔS), and free energy (ΔG) were determined by fitting the integrated titration data using one set of sites fitting model by a nonlinear least-squares method implemented in MicroCal ITC200 analysis software Origin 7.0 (Malvern, www.microcal.com). The standard errors are fitting errors of the ITC titration curves.

### PBM assay

For PBM assay, the BEN domain of human BANP (aa 208-393) was subcloned into a pET28-GST-linker vector and expressed using PURExpress *In Vitro* Protein Synthesis Kit (New England BioLabs) to produce an N-terminal GST-tagged recombinant protein. PBM experiments were performed as previously described (22). Briefly, the binding specificity of the human BANP BEN domain protein was analyzed in duplicates using two types of arrays (HK and ME) (25, 26). The HK array was designed by Hilal Kazan using the methodology described by Philippakis *et al* (26), while the ME array was developed by Julian Mintseris and Mike Eisen (25). Each array consists of ∼41,000 60-base probes with completely different sequences. Each PBM sequence library is designed according to the de Bruijn method, such that all possible 10-mers and 32 copies of every nonpalindromic 8-mer are included in each array, guaranteeing an unbiased estimate of protein binding preference. E-scores are rank-based, nonparametric enrichment statistics, and Z-scores are significance estimates based on the normal distribution of intensities. The sequence logos with E-score >0.45 and Z-score >6 from the PBM experiments were considered as specific binding (22, 27). The position weight matrices for the preferential sequence of the BANP BEN domain were generated by the position weight matrix_align algorithm using the sequence containing the highly correlated E- and Z-scores (22).

### FP assay

FP assay was carried out to monitor the interactions of the BEDN6 BEN domain proteins with fluorescently labeled DNAs in solution. The 5′ fluorescein amidites-labeled DNA was synthesized by General Biosystems Co Ltd and annealed into DNA duplexes as described for the ITC assay. FP binding assay was performed with a constant DNA concentration of 40 nM and a varied concentration of the BEN domain protein ranging from low to high micromolar in a buffer containing 20 mM Tris-HCl (pH 7.5), 150 mM NaCl, 1 mM DTT, and 0.01% Triton X-100. FP signal was analyzed by Synergy H1 multimode reader (BioTek) with excitation and emission wavelengths of 485 nm and 528 nm, respectively. We did two triplicates for each sample. The *K*_d_ values were obtained by fitting the data using one site-binding model by the Nonlinear Curve fitting (Origin 2021). The error for each *K*_d_ value is the fitting error, and the experimental error is based on the average value of the replicates.

### Differential scanning fluorometry assay

The stability of protein mutants was analyzed using differential scanning fluorometry assay. The protein samples were used at a final concentration of 0.8 mg/mL in a buffer with 20 mM Tris-HCl (pH 7.5) and 150 mM NaCl, and the fluorescent dye SYPRO Orange (Sigma) was used at a final concentration of 8X. To measure the melting curves of protein samples, the temperature was increased from 25 °C to 90 °C with a gradual fate of 2 °C/min using the Real-time PCR instrument (Light Cycler 480, Roche). The excitation and emission wavelengths were 465 nm and 580 nm, respectively. Melting curves were analyzed using Graphpad, and Tm values for each sample are calculated based on three independent experiments.

### Protein crystallization

Crystallization was carried out using the sitting drop vapor diffusion method at 18 °C by mixing equal volumes (0.5 μL) of protein solution and reservoir solution. Crystals of the BANP BEN domain (16 mg/mL) in apo form were grown in a reservoir solution containing 20% PEG500∗MME (v/v), 10% PEG 20000 (w/v), 0.09 M halogens, 0.1 M Tris-base, and BICINE (pH 8.5). To obtain crystals of the BEN-DNA complex, the BANP BEN domain protein (10 mg/mL) and BEND6 BEN domain protein (20 mg/mL) were mixed with different DNA oligos at a molar ratio of 1:1.5 or 1:1.2, respectively. Crystals of the BANP-CGCG DNA complex were grown in a reservoir solution containing 20% PEG 3350 (w/v), 0.02 M sodium/potassium phosphate, and 0.1 M Bris-tris propane (pH 6.5). Crystals of the BANP-mCGCG DNA complex were grown in a reservoir solution consisting of 0.2 M sodium fluoride, 0.1 M BTP-tris (pH 6.5), 20 % PEG 3350 (w/v). Crystals of the BEND6-CGCG DNA complex were grown in a reservoir solution consisting of 0.12 M ethylene glycol, 0.1 M sodium Hepes, 0.1 M MOPS (acid), 12.5% 2-methyl-2,4-pentanediol (v/v), 12.5% PEG 1000 (v/v), and 12.5% PEG 3350 (w/v). Crystals of the BEND6-CGmCG DNA complex grown in a reservoir solution consisting of 0.1 M carboxylic acids, 0.1 M imidazole, 0.1 M Mes monohydrate (acid) (pH 6.5), 12.5% 2-methyl-2,4-pentanediol (v/v), 12.5% PEG 1000 (v/v), and 12.5% PEG 3350 (w/v).

### Data collection and structure determination

Prior to diffraction data collection, the crystals were soaked in a cryoprotectant consisting of their respective reservoir solution supplemented with an additional 20% (v/v) ethylene glycol or glycol and then flash-cooled into liquid nitrogen. Diffraction data of the BANP BEN domain were collected at SSRF 18U beamline at 100 K and then processed with the HKL 2000 suite (28) and Collaborative Computational Project, Number 4 (29). Diffraction data of the BANP-mCGCG complex were collected at SSRF 10U2 beamline at 100 K and then processed with the X-ray Detector Software (https://xds.mr.mpg.de/) (30) and Collaborative Computational Project, Number 4 (29). The diffraction data of the BEND6-DNA complex were collected and processed using an in-house Bruker METALJET at 100 K. The structure of the BANP BEN domain in apo form was solved by the molecular replacement method implemented in the program PHASER (31) using the BANP BEN domain structural model from the AlphaFold2 Protein Structure Database (32, 33) as the search model. The BANP-DNA and BEND6-DNA structures were solved by the molecular replacement method with the BANP BEN structure as the search model. Model building was performed with Coot (https://www.ccp4.ac.uk/download/#os) (34), and structure refinement was performed with REFMAC (35) and phenix.refine (36). Statistics of the diffraction data, the structure refinement, and the quality of the final structure models are summarized in Table S1.

## Data availability

The crystal structures of the BEN domain of BANP in apo form and in complex with unmethylated and methylated DNA and the BEN domain of BEND6 in complexes with unmethylated and methylated DNAs have been deposited in the Protein Data Bank under accession codes 7YUG, 7YUK, 8HTX, 7YUL, and 7YUN, respectively.

## Supporting information

This article contains supporting information.

## Conflict of interest

The authors declare that they have no conflicts of interest with the contents of this article.
