# Supplementary material for: Structural insights into DNA recognition by the BEN domain of the transcription factor BANP
Source: J Biol Chem. 2023 Apr 20;299(6):104734. doi: 10.1016/j.jbc.2023.104734 (PMC10206803; doi:10.1016/j.jbc.2023.104734)
Supplement: Table S1 [file mmc1.docx]

**Table S1. Data collection and refinement statistics**

| **Structure** | **BANP**  **(aa 208-324)** | **BANP-DNA**  **(aa 208-347)** | **BANP-mCGCG DNA**  **(aa 208-347)** | **BEND6-DNA**  **(aa 170-271)** | **BEND6-CGmCG DNA**  **(aa 170-271)** |
| --- | --- | --- | --- | --- | --- |
| **PDB ID** | **7YUG** | **7YUK** | **8HTX** | **7YUL** | **7YUN** |
| **DNA sequences** |  | 5’-CTCTCGCGAGAG-3’  3’-GAGAGCGCTCTC-5’ | 5’-CTCTmCGCGAGAG-3’  3’-GAGAGCGmCTCTC-5’ | 5’-CTCTCGCGAGAG-3’  3’-GAGAGCGCTCTC-5’ | 5’-CTCTCGmCGAGAG-3’  3’-GAGAGmCGCTCTC-5’ |
| **Diffraction data** |  |  |  |  |  |
| Space group | *P*2_1_2_1_2_1_ | *P*2_1_2_1_2 | *P*2 2_1_2 | *P*2_1_2_1_2 | *P*2_1_ |
| Cell dimensions |  |  |  |  |  |
| *a, b, c* (Å) | 39.65, 50.04, 54.09 | 36.97, 129.90, 40.13 | 42.35, 92.65, 127.02 | 69.09, 39.51, 43.72 | 39.48, 67.28, 53.02 |
| *α, β, γ* (°) | 90, 90, 90 | 90, 90, 90 | 90, 90, 90 | 90, 90, 90 | 90, 100.72, 90 |
| Resolution (Å) | 50.00-1.10 (1.12-1.10)^a^ | 50.00-2.11 (2.17-2.11) | 38.54-2.80 (2.95-2.80) | 27.11-1.82 (1.85-1.82) | 34.32-2.13 (2.16-2.13) |
| Completeness (%) | 99.9 (99.6) | 99.9 (99.4) | 99.7 (99.9) | 99.4 (92.7) | 99.9 (99.8) |
| R_merge_^b^ | 0.096 (0.285) | 0.086 (0.471) | 0.124 (0.740) | 0.045 (0.159) | 0.056 (0.240) |
| I/σI | 45.5 (9.9) | 17.0 (4.5) | 12.6 (3.8) | 34.2 (9.8) | 17.4 (4.5) |
| CC1/2 | 0.995 (0.974) | 0.998 (0.978) | 0.995 (0.979) | 0.999 (0.974) | 0.999 (0.924) |
| Redundancy | 12.4 (12.3) | 12.4 (12.2) | 10.8 (11.6) | 7.7 (3.3) | 4.8 (3.1) |
| **Refinement** | |  |  |  |  |
| Resolution (Å) | 32.00-1.10 | 40.17-2.11 | 31.76-2.80 | 27.11-1.82 | 34.32-2.13 |
| Reflections (working set) | 42104 | 11149 | 12691 | 10599 | 14571 |
| Reflections (test set) | 2348 | 615 | 646 | 567 | 780 |
| No. atoms/B-factor (Å^2^) | 1206/11.54 | 1496/40.69 | 2882/81.26 | 1144/16.86 | 2108/24.34 |
| Protein | 1014/11.00 | 955/42.33 | 953/81.55 | 756/17.83 | 733/25.85 |
| DNA |  | 243/46.69 | 244/80.70 | 243/14.04 | 244/24.05 |
| Water | 189/25.64 | 55/39.46 |  | 140/26.42 | 155/29.64 |
| R_work_/R_free_^c^ | 0.124/0.145 | 0.226/0.261 | 0.232/0.272 | 0.158/0.194 | 0.184/0.233 |
| RMS deviations |  |  |  |  |  |
| Bond lengths (Å) | 0.019 | 0.012 | 0.004 | 0.011 | 0.009 |
| Bond angles (°) | 1.87 | 1.48 | 0.64 | 1.83 | 1.65 |

| ^a^ Numbers in parentheses represent the highest resolution shell. | | |
| --- | --- | --- |
| ^b^ *R*_merge_ = ∑*_hkl_*∑*_i_*\|*I_i_*(*hkl*)−<*I*(*hkl*)>\|/∑*_hkl_*∑*_i_I_i_*(*hkl*). | |  |
| ^c^ *R*-factor = ∑*_hkl_*\|\|*F_o_*\|−\|*F_c_*\|\|/∑*_hkl_*\|*F_o_*\|. |  |  |
