# Supplementary material for: Structural insights into DNA recognition by the BEN domain of the transcription factor BANP
Source: J Biol Chem. 2023 Apr 20;299(6):104734. doi: 10.1016/j.jbc.2023.104734 (PMC10206803; doi:10.1016/j.jbc.2023.104734)
Supplement: Supplemental Figures [file mmc2.pdf]

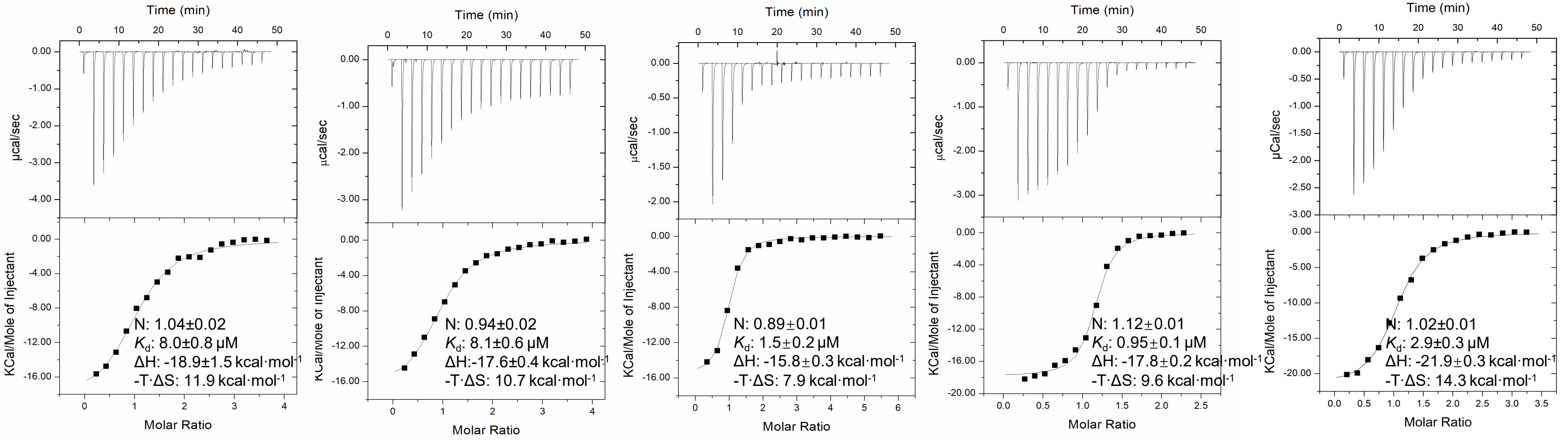

BANP BEN-CGCG DNA (repeat)
5'-CTC**TCGCG**AGAG-3'

BANP BEN-CGCG DNA (repeat)

BANP BEN-AAATCTCG (repeat)
5'-GCAAA**TCTCG**TA-3'

BANP BEN-AAATCTCG (repeat)
5'-GCAAA**TCTCG**TA-3'

BANP BEN-DNA
5'-GCcag**TCTCG**TA-3'

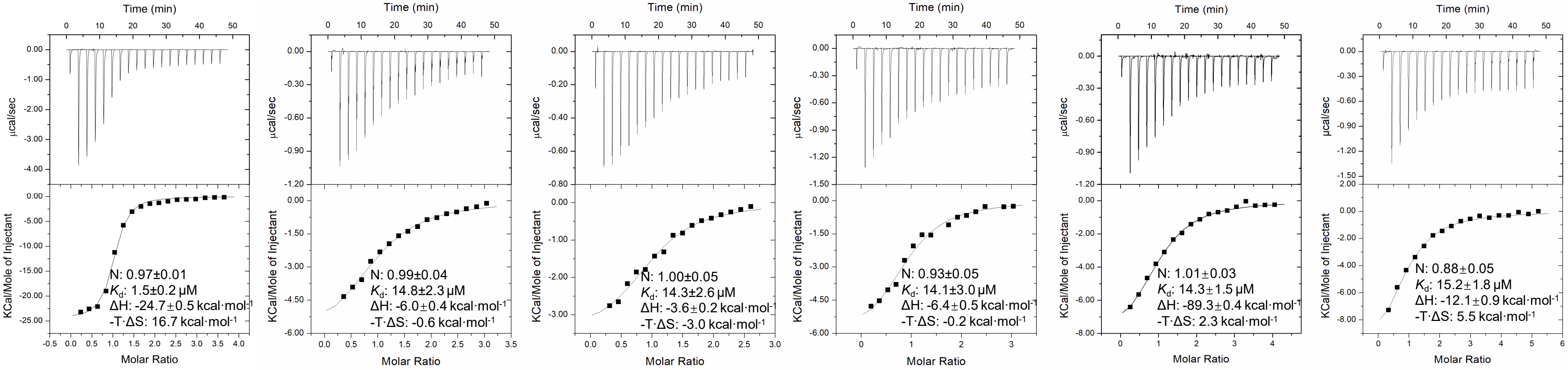

BANP BEN-DNA
5'-GCAAA**TCGCG**TA-3'

BANP BEN-DNA
5'- CTC**tggcg**AGAG -3'

BANP BEN-DNA
5'- CTC**acgcg**AGAG-3'

BANP BEN-DNA
5'-CTC**gagcg**AGAG-3'

BANP BEN-DNA (repeat)
5'-GC**cagcaaca**TA-3'

BANP BEN-DNA (repeat)
5'-GC**cagcaaca**TA-3'

Figure S1. The ITC binding curves of the BEN domain of BANP to different DNA based on the identified consensus motifs. The mutated nucleotides are shown in lower case. Only one strand of the DNA duplex is shown. The errors are fitting errors of the ITC titration curves.

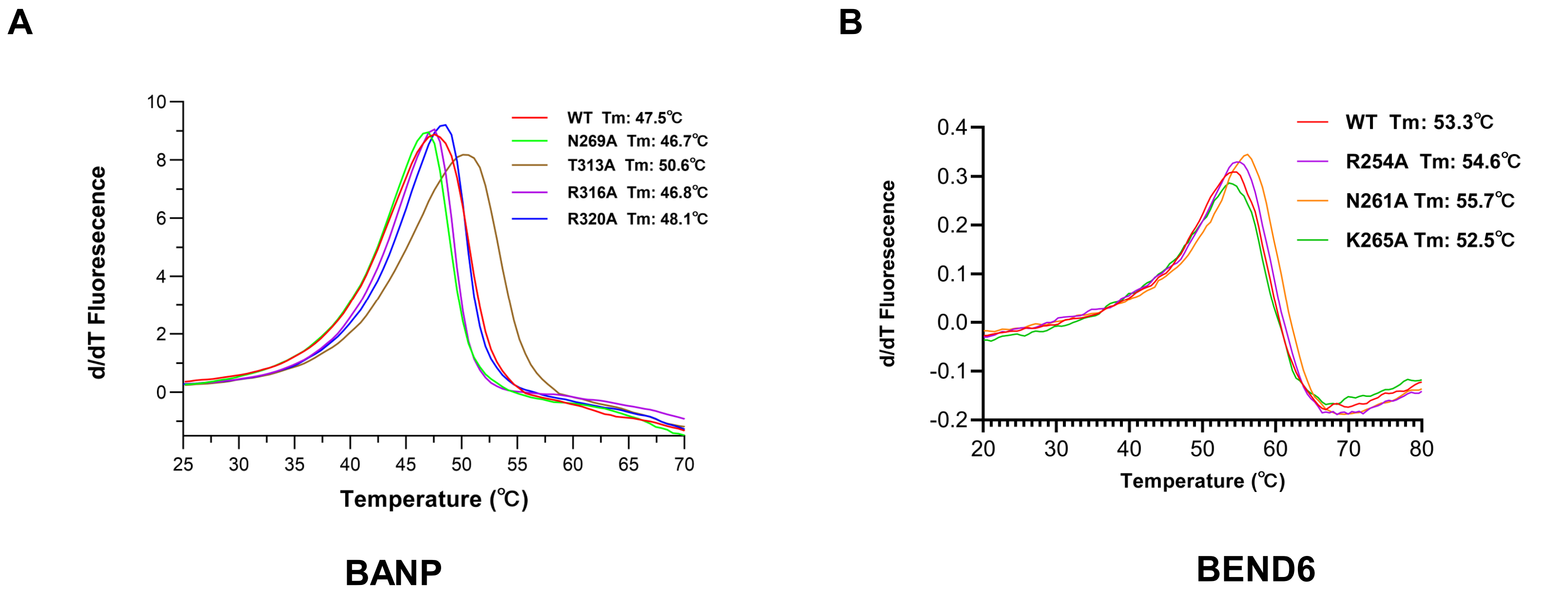

Figure S2. Stability analysis of mutant proteins using differential scanning fluorometry (DSF).

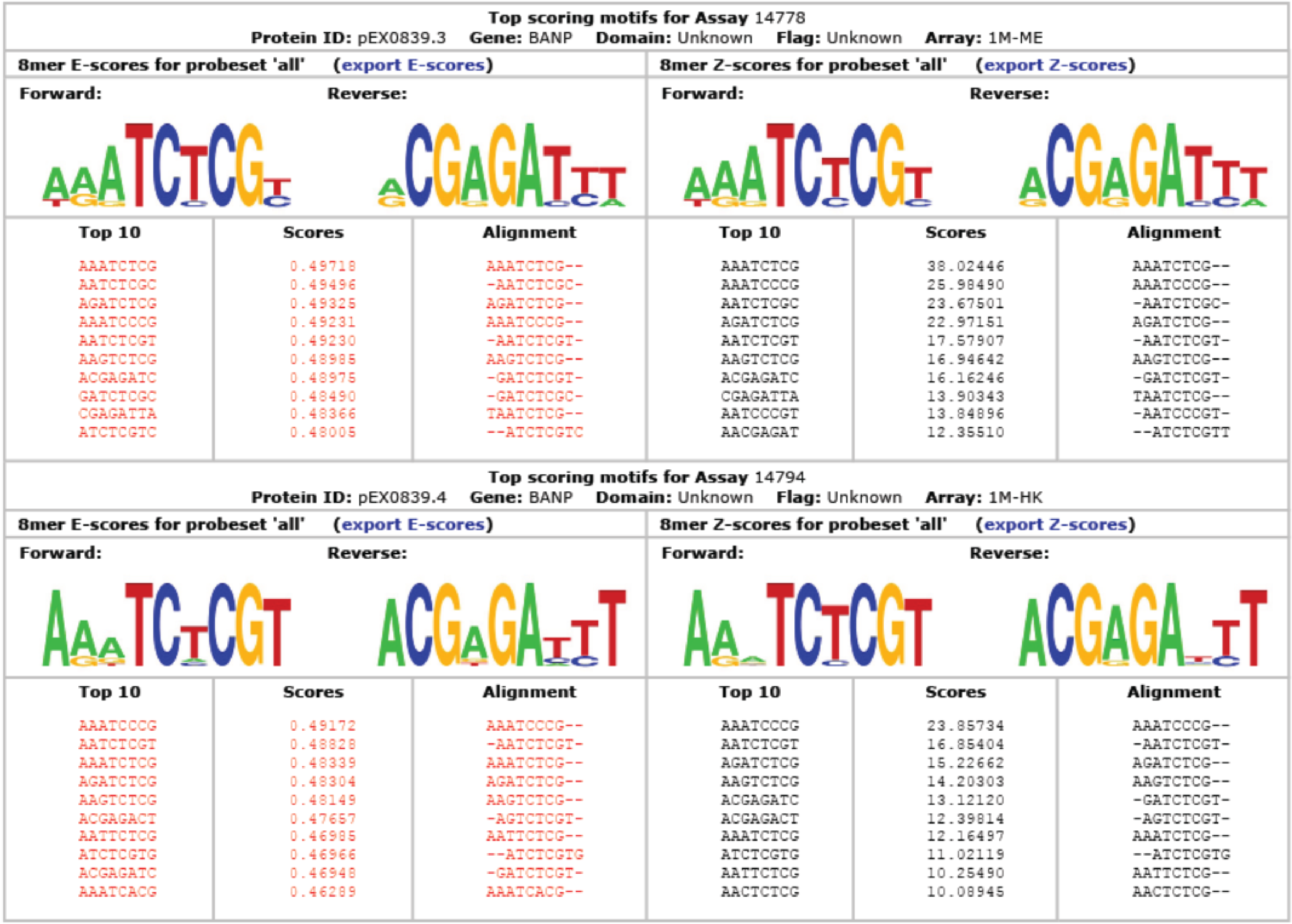

Top scoring motifs for Assay 14778

Protein ID: pEX0839.3 Gene: BANP Domain: Unknown Flag: Unknown Array: 1M-ME

8mer E-scores for probeset 'all' (export E-scores)

Forward: Reverse:

Top 10	Scores	Alignment
AAATCTCG	0.49718	AAATCTCG--
AATCTCGC	0.49496	-AATCTCGC-
AGATCTCG	0.49325	AGATCTCG--
AAATCCCG	0.49231	AAATCCCG--
AATCTCGT	0.49230	-AATCTCGT-
AAGTCTCG	0.48985	AAGTCTCG--
ACGAGATC	0.48975	-GATCTCGT-
GATCTCGC	0.48490	-GATCTCGC-
CGAGATTA	0.48366	TAATCTCG--
ATCTCGTC	0.48005	--ATCTCGTC

8mer Z-scores for probeset 'all' (export Z-scores)

Forward: Reverse:

Top 10	Scores	Alignment
AAATCTCG	38.02446	AAATCTCG--
AAATCCCG	25.98490	AAATCCCG--
AATCTCGC	23.67501	-AATCTCGC-
AGATCTCG	22.97151	AGATCTCG--
AATCTCGT	17.57907	-AATCTCGT-
AAGTCTCG	16.94642	AAGTCTCG--
ACGAGATC	16.16246	-GATCTCGT-
CGAGATTA	13.90343	TAATCTCG--
AATCCCGT	13.84896	-AATCCCGT-
AACGAGAT	12.35510	--ATCTCGTT

Top scoring motifs for Assay 14794

Protein ID: pEX0839.4 Gene: BANP Domain: Unknown Flag: Unknown Array: 1M-HK

8mer E-scores for probeset 'all' (export E-scores)

Forward: Reverse:

Top 10	Scores	Alignment
AAATCCCG	0.49172	AAATCCCG--
AATCTCGT	0.48828	-AATCTCGT-
AAATCTCG	0.48339	AAATCTCG--
AGATCTCG	0.48304	AGATCTCG--
AAGTCTCG	0.48149	AAGTCTCG--
ACGAGACT	0.47657	-AGTCTCGT-
AATTCTCG	0.46985	AATTCTCG--
ATCTCGTG	0.46966	--ATCTCGTG
ACGAGATC	0.46948	-GATCTCGT-
AAATCACG	0.46289	AAATCACG--

8mer Z-scores for probeset 'all' (export Z-scores)

Forward: Reverse:

Top 10	Scores	Alignment
AAATCCCG	23.85734	AAATCCCG--
AATCTCGT	16.85404	-AATCTCGT-
AGATCTCG	15.22662	AGATCTCG--
AAGTCTCG	14.20303	AAGTCTCG--
ACGAGATC	13.12120	-GATCTCGT-
ACGAGACT	12.39814	-AGTCTCGT-
AAATCTCG	12.16497	AAATCTCG--
ATCTCGTG	11.02119	--ATCTCGTG
AATTCTCG	10.25490	AATTCTCG--
AACTCTCG	10.08945	AACTCTCG--

Figure S3. **Results from the protein binding microarrays.** The sequences with E-score more than 0.45 and Z-score above 6 from the PBM experiment are considered as specific binding sequences, and the top ten sequences from both ME and HK assays are shown, respectively.

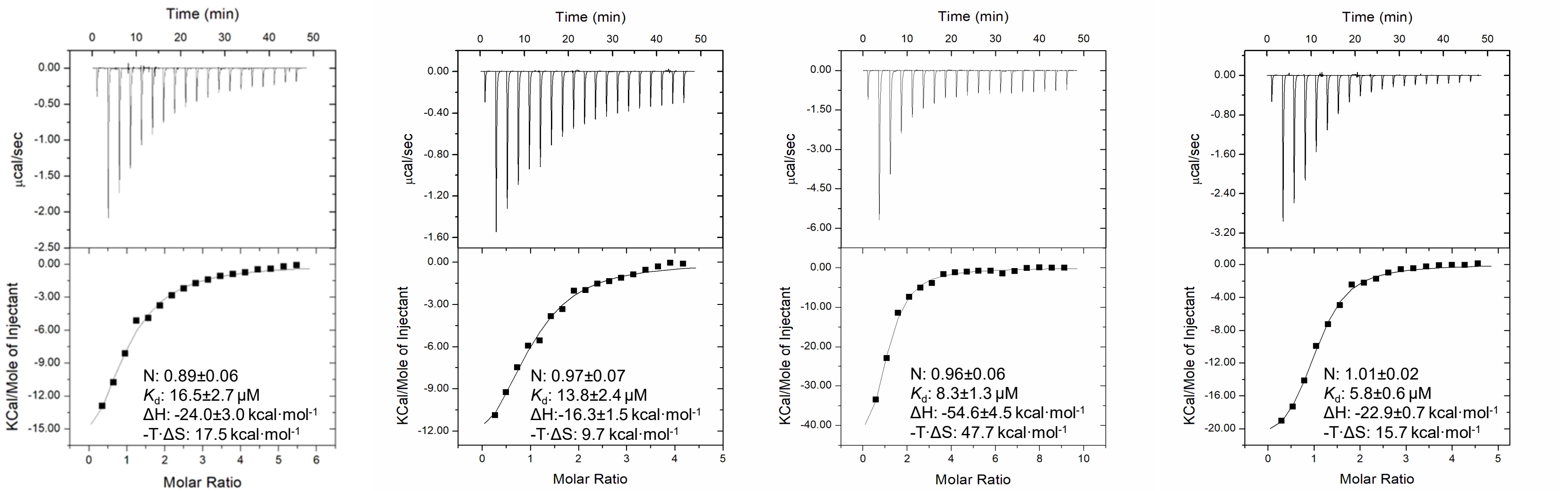

BANP BEN-mCGCG (repeat) 5'-CTC**TmCGCG**AGAG-3'

BANP BEN-mCGCG (repeat) 5'-CTC**TmCGCG**AGAG-3'

BANP BEN-CGmCG (repeat) 5'-CTC**TCGmCG**AGAG-3'

BANP BEN-CGmCG (repeat) 5'-CTC**TCGmCG**AGAG-3'

Figure S4. ITC binding repeats of the BANP BEN domain to different methylated DNA. Only one strand of the DNA duplex is shown. The errors are fitting errors of the ITC titration curves.

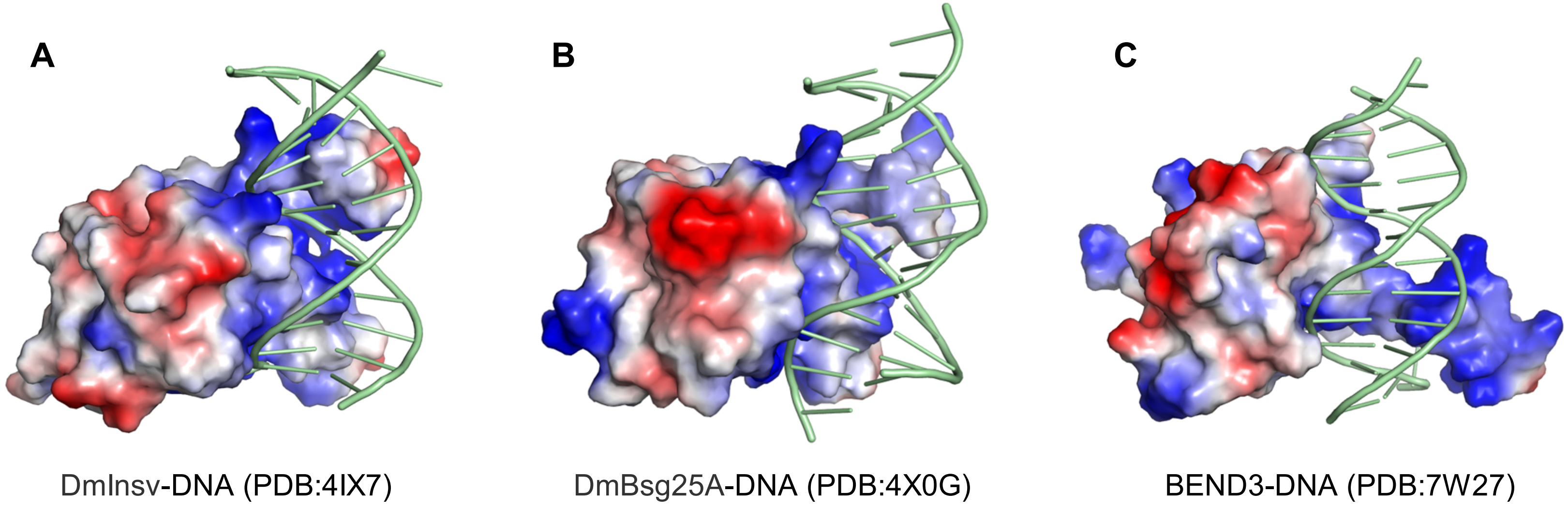

Figure S5. Electrostatic surface potential representation of the BEN domains of DmInsv, DmBsg25A and human BEND3 BEN4 in complex with their target DNAs. Electrostatic potential was calculated by PyMOL.
